# Intense pulsed light (IPL) annealed sol–gel derived ZnO electron injector for the production of high efficiency inverted quantum dot light emitting devices (QLEDs)

**DOI:** 10.1039/c8ra08136k

**Published:** 2018-10-30

**Authors:** Poopathy Kathirgamanathan, Muttulingam Kumaraverl, Raghava Reddy Vanga, Seenivasagam Ravichandran

**Affiliations:** Organic Electronics Group, Wolfson Centre, Brunel University London Uxbridge UB8 3PH UK p.kathir@brunel.ac.uk

## Abstract

Room temperature intense pulsed light annealing (photonic annealing, pulsed forge) renders the sol–gel derived ZnO films highly conductive and hydrophobic with improved interface with the colloidal quantum dots. The IPL annealed ZnO proved to be a better electron transporter/injector in inverted devices with QDs. Both the current and power efficiencies of red devices comprising IPL annealed ZnO were 13.75 and 37.5 fold higher than the identical devices produced with thermally annealed ZnO. The lifetime of the devices with IPL annealed ZnO was found to be fivefold longer than the thermally annealed ZnO counterpart. Thermally aged devices comprising IPL annealed ZnO gave a maximum current efficiency of 23 cd A^−1^ and a power efficiency of 30 lm W^−1^.

## Abstract 2

Room temperature intense pulsed light annealing (photonic annealing, pulsed forge) renders the sol–gel derived ZnO films highly conductive and hydrophobic with improved interface with the colloidal quantum dots.

## Graphical abstract

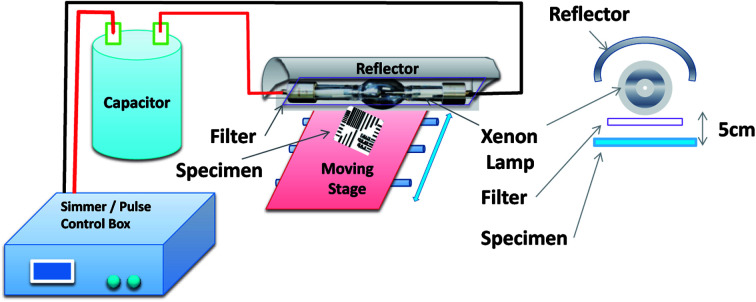

## Introduction

The recent trend towards flexible and rollable displays, flexible printed electronics and electronic textiles demand the use of polymeric films because of their attractive mechanical properties and low cost.^1^ For example, polyester film (PET, polyethylene terephthalate) has a very low glass transition temperature (*T*_g_ = 81 °C) and therefore low temperature processing methods are essential. Fabrication of devices on flexible substrates (*e.g.* PET) such as plastics requires low temperature processing of below 80 °C. Thus, conventional thermal annealing/curing/sintering cannot therefore be performed on these substrates. Printed copper inks and silver inks have to be sintered (annealed) in order for them to become conducting. The typical method employed for this purpose is called intense pulse light from a flash lamp (also called flash lamp annealing, photonic annealing or Pulse Forge) using a high intensity xenon lamp emitting light from 250 nm to 1150 nm. The curing and the annealing time can vary from 0.1 ms to 10 ms. The speed of the annealing enables roll to roll printing and annealing of conductive tracks.

Intense Pulsed Light (Pulse Forge) is an attractive technique as the transient heating (1 μs to 10 ms pulse, a non-equilibrium annealing process) is carried out on a low temperature substrate. It is possible for the substrate to attain a significantly higher temperature than the substrate can ordinarily withstand under an equilibrium heating source such as an oven or hotplate. It is only the surface that heats up rather than the substrate. Since the rate of thermal curing process (drying/sintering/annealing) generally increase exponentially with temperature, this process allows materials to be dried much more rapidly (<2 ms) than with a conventional oven. Furthermore, it enables the creation of new materials, surfaces, morphologies and structural modifications, creating material set which are not feasible with the conventional thermal methods.^2,3^

IPL is routinely employed in annealing conductive silver and copper inks for electronic applications.^4,5^ However, its potential application in other areas where rapid drying/annealing (100 μs to 10 ms) can be applied with beneficial surface effects (physical/chemical/morphological) is still being explored in metal oxide semiconductor devices, photovoltaics, printed electronics, flexible electronics^6–10^

**Fig. 1:**
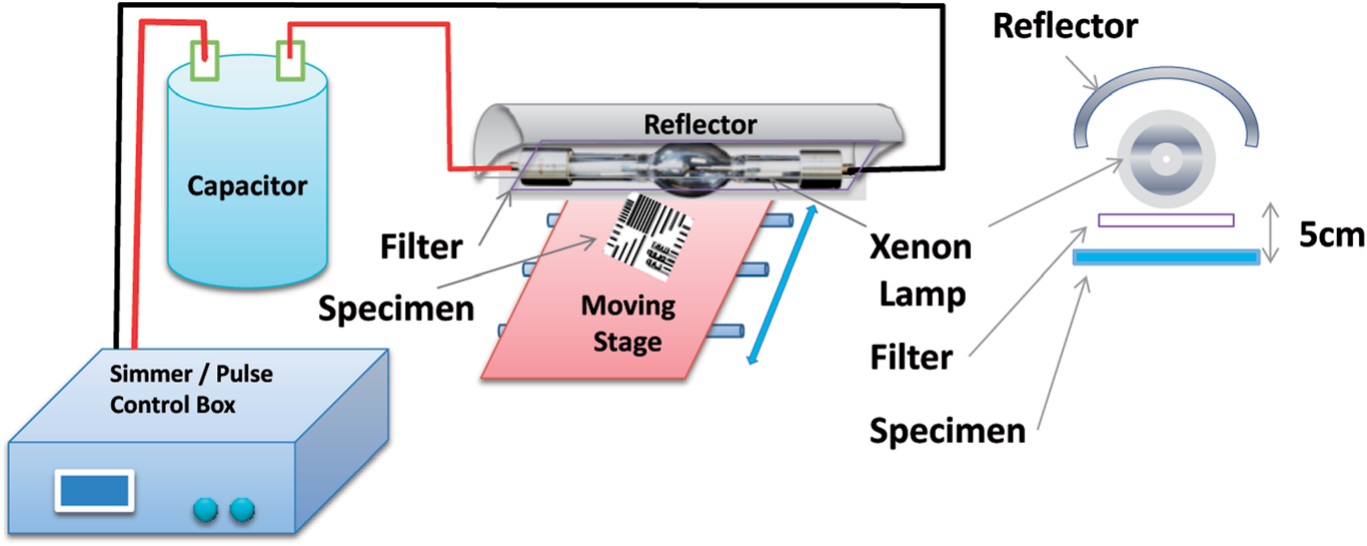
The experimental set up for irradiation of the substrate with Intense Pulse Light (IPL, Pulse Forge).

Fig. 1 shows a schematic of an IPL system (*e.g.* Novacentrix, Pulse Forge 1300 used in this work). The IPL device consists of a Xenon flash lamp, a reflector, a power supply, capacitors and a pulse controller. The pulse controller triggers the capacitor which delivers an electrical current pulse of predetermined time (typically, milliseconds) generating light pulses to irradiate the substrate. Intensity and the energy density can be controlled by the applied voltage, pulse duration, number of pulses and the free temporal period.

**Fig. 2:**
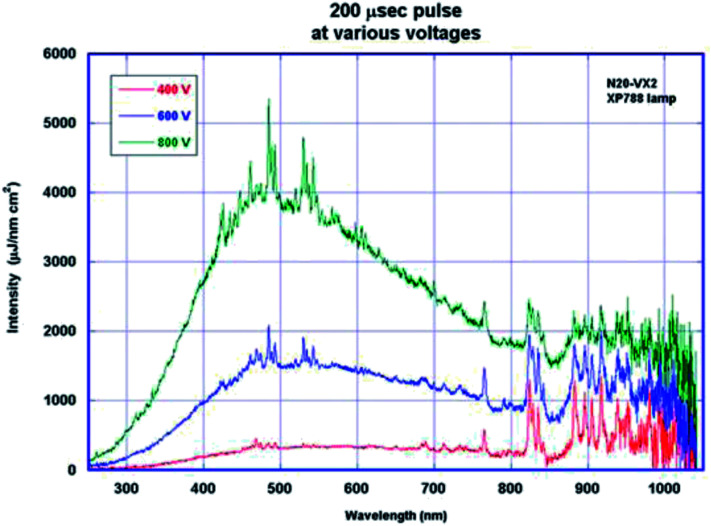
Spectral output from the IPL (Pulse Forge, Novacentrix).

The emission spectrum of a Xenon lamp is primarily dependent on the applied current density. As the voltage is increased, the intensity of light increases more markedly in the visible region (400 nm–800 nm) as shown in Fig. 2.

IPL has been utilised to convert highly resistive ZnO films produced by chemical bath deposition method from 1 × 10^6^ ohms per square to conducting (40 ohms per square) and transparent (90% in the visible range) simply by irradiating with a Xenon lamp (3000 V, 2 ms pulse, pulse number, 50, rest time between the pulses, 3 s).^10^ The possible mechanism for the conductivity increase is still not fully understood, but photodoping was suggested as the main reason.^10^ Organic ligands in IGZO (Indium Gallium Oxide precursors) can be removed by IPL in milliseconds and the resultant IGZO has much higher mobility than the thermally annealed counterpart.^25^

Further, photonic curing allows large scale processing (batch to batch, 200 mm × 200 mm) and roll to roll processing where the annealing is carried out in millisecond time scale. This process is now commonly used in sintering printed copper and silver inks on paper or plastic substrates^11^ where the solvents and the organic binders are removed by NIR-UV (portion) and UV-VIS portion promotes the sintering from the high intensity light pulse lamps.

Colloidal quantum dot light emitting devices have become one of the most exciting candidates for the next generation displays and solid state lighting as they offer colour tunability simply by changing the size and they are solution processable by spin coating, inkjet printing, slot coating or spray coating.^12–21,26^ Colloidal semiconductor nanocrystals (quantum dots, QDs) are composed of an inorganic core (made up of a few hundred to a few thousand atoms) surrounded by an inorganic shell, which in turn is surrounded by an organic surfactant molecules (ligands). Typical quantum dots^20^ include CdSe/ZnS, InP/ZnS, perovskites and CuInS_2_. Research on electroluminescence of colloidal quantum dots is gathering momentum as the colour requirement for Rec 2020 (ITU-R-BT2020) cannot be achieved by means of state of art OLED materials^19^ with simple device architectures. The usual OLEDs based on fluorescent, phosphorescent and thermally activated delayed fluorescent emitters only achieve roughly 90% of NTSC standards.^21–26^

QLEDs can be fabricated either as a conventional device (*e.g.* ITO (anode)/hole injector/hole transporter/QD/electron transporter/electron injector/Al) or inverted (*e.g.* ITO (cathode)/electron injector/electron transporter/QD/hole transporter/hole injector/Al).^26^ Usually, the solvent processable hole injector employed in a conventional QLEDs is poly(3,4-ethylenedioxythiophene)polystyrene (PEDOT-PSS). Long term stability (life-time) is a problem with PEDOT-PSS because it is hygroscopic and acidic. In the inverted QLEDs, n-type metal oxides such as ZnO, TiO_2_ and ZrO_2_ have been employed as they have the advantage of being air stable and unaffected by the organic solvents in which the QDs are solubilised. Considerable amount of work is on-going on surface treatment of the electron injecting metal oxides and hole injecting metal oxides(*e.g.* MoO_3_ and NiO) to achieve high efficiency and long life time.^31^

ZnO is a very effective electron injector and transporter owing to it high electron mobility (1 × 10^−3^ to 1 × 10^2^ cm^2^ V^−1^ s^−1^ depending on the method of production) and hence injects excess electrons into the QDs and the devices degrade owing to high leakage current.^26^ This also leads to charged excitons resulting in poor electroluminescent efficiencies owing to Auger recombination. One of the ways to solve this issue is by reducing the excess electrons by inserting wide bandgap barrier between the ZnO and the QD layer such as a thin layer of PMMA, PEI or Al_2_O_3_. To achieve the best performance, it has been claimed that the ZnO-NP used in the QLED devices have to be below 5 nm.^32–34^ Aluminium, Mg, Li and Cs doped ZnO were also found to be efficient electron injectors for QLEDs and perovskite QLEDs.^35–38^

We have been interested in QLEDs for the past four years^17,19,21,26^ where the ZnO derived from sol–gel were employed as the electron injector/electron transporter which requires 30–60 minutes of thermal annealing at 120 °C or above. We were interested in exploring if the ZnO derived from sol–gel can be annealed by IPL in millisecond time scale without any deterioration in the device characteristics and indeed if any improvement can be obtained. Further, we wanted to develop low temperature annealing processes that could be used in roll to roll production of QLEDs. We demonstrate here that IPL can successfully be employed to produce high efficiency QLEDs. The efficiency of QLEDs so produced can be further dramatically enhanced by thermal aging (baking) of the devices at 40 °C in a similar manner to devices comprising thermally annealed ZnO as the electron transporter/injector.

## Experimental

### 1. Synthesis of ZnO nanoparticles (ZnO-M1)

Zn(OAc)_2_·2H_2_O (0.22 g, 0.1 M), monoethanolamine (0.06 g, 0.1 M) and anhydrous ethanol (10 ml) were added to a round bottom flask and refluxed for 1 h to form a clear homogeneous solution of ZnO-M1.

### 2. Fabrication of ZnO film

This was spin coated at 3000 rpm onto a patterned ITO (100 mm × 100 mm) and annealed at either 60 °C (vacuum, 30 minutes), 120 °C (vacuum, 30 minutes) or 60 °C (vacuum, 30 minutes) + IPL (Pulse Forge, at room temperature and in air) depending on the device architecture. In the last case, the ZnO films annealed (vacuum) in vacuum at 60 °C (this is done so as to remove all the solvents as otherwise it damages the xenon lamps and its associated electronics) and then annealed under intense light pulse (Photonic Curing Machine, Pulse Forge 1300, Nova Centrix, Texas, USA) at three selected voltages, namely, 450 V, 650 V and 850 V respectively (Table 1).

**Table 1:** Experimental parameters for Intense Pulsed Light (IPL-Pulse Forge)

Voltage	Pulse width/ms	No. of pulses	Total exposure time/ms	Total energy density/J cm^−2^
450 V	1.5	5	7.5	22.6
650 V	1.5	3	4.5	37.8
850 V	1.5	2^a^	3.0	23.6

a No of pulses limited by the capability of the equipment.

### 3. Device fabrication

QD films (25 nm) were produced by spin coating (3000 rpm) a 10 mg ml^−1^ solution of CdSe/ZnS quantum dot solution in toluene and dried at 80 °C in vacuum.

All other QLED layers, TCTA (Tris(4-carbazolyl)-9-ylphenyl)amine) and α-NPB (*N*,*N*′-di(1-naphthyl)-*N*,*N*′-diphenyl-(1,1′-biphenyl)-4,4′-diamine) were all vacuum evaporated (1 A s^−1^) except for HATCN (1,4,5,8,9,11-hexaazatriphenylene hexacarbonitrile) which was evaporated at 0.5 A s^−1^ in an ULVAC OLED plant (Solciet, purchased from ULVAC, Japan) as described in our previous papers (see for example, ref. 19, 26 and 27. Devices were fabricated using ULVAC Solciet thin film vacuum deposition equipment purchased from ULVAC, Japan. The hole transporting and hole transporting layers were deposited sequentially onto 100 cm × 100 cm ITO coated glass substrates under a vacuum of 10^−5^ torr at a deposition rate of 1 Å s^−1^. Deposition of the cathode (Al) also took place under vacuum ahead of device encapsulation in a nitrogen-filled glove box.

### 4. Thin film characterisation

Absorption characteristics of the QDs were analysed by UV-VIS spectroscopy (Shimadzu UV-1800 UV Spectrophotometer) and photoluminescent (PL) emission spectra were collected by a spectrophotometer (Perkin Elmer LS-55 Luminescence Spectrometer). Electroluminescent (EL) characterisation was carried out using a Minolta CS-1000 Spectroradiometer as previously published by Kathirgamanathan *et al.*^27^ Work function measurements were performed by photoelectron spectroscopy on either AC-II or AC-III (manufactured by Riken Keiki Co Ltd, Japan) as appropriate.

Capacitance measurements were carried out on thin QD films (100 nm) using an impedance analyser (Hewlett Packard 4284 A). Electron only devices of ZnO films were made where the electrodes were Al (ITO/Al (100 nm)/ZnO (135 ± 5 or 35 ± 5 nm)/Al (100 nm)).

Surface topology of the various ZnO films was examined by AFM (Digital Instruments, Dimension 3100) which was also utilized to determine their thicknesses in addition to Nanosurf (Leicester, UK) and TEM (Jeol JEM-2100F) was used to work out the particle size. Full scale nano's software (USA) was employed to work out the average particle size and their distribution. SEM (Supra 35 V with EDAX) was performed to determine surface structure of ZnO films and their composition.

X-ray powder diffraction was performed on Bragg–Brentano Bruker D8 Advance diffractometer equipped with copper tube and LynxEye position sensitive detector (Cu Kα radiation (wavelength of 1.5406 Å from a generator operating at 40 keV and 40 mA)).

### 5. Device architecture

We fabricated devices based CdSe/CdS/ZnS quantum dots (toluene) as follows:

Set A: ITO/ZnO (NP) (40 nm, ZnO-M1, 60 °C)/Red-QD-X (25 nm)/TCTA (10 nm)/HTL (α-NPB (20 nm)/HATCN (10 nm)/Al).

Set B: ITO/ZnO (NP) (40 nm, ZnO-M1, 120 °C)/Red-QD-X (25 nm)/TCTA (10 nm)/HTL (α-NPB (20 nm)/HATCN (10 nm)/Al).

Set C: ITO/ZnO (NP) (40 nm, ZnO-M1, 60 °C) and Intense Light Pulse (ILP)/Red-QD-X (25 nm)/TCTA (10 nm)/HTL (α-NPB (20 nm)/HATCN (10 nm)/Al).

**Fig. 3:**
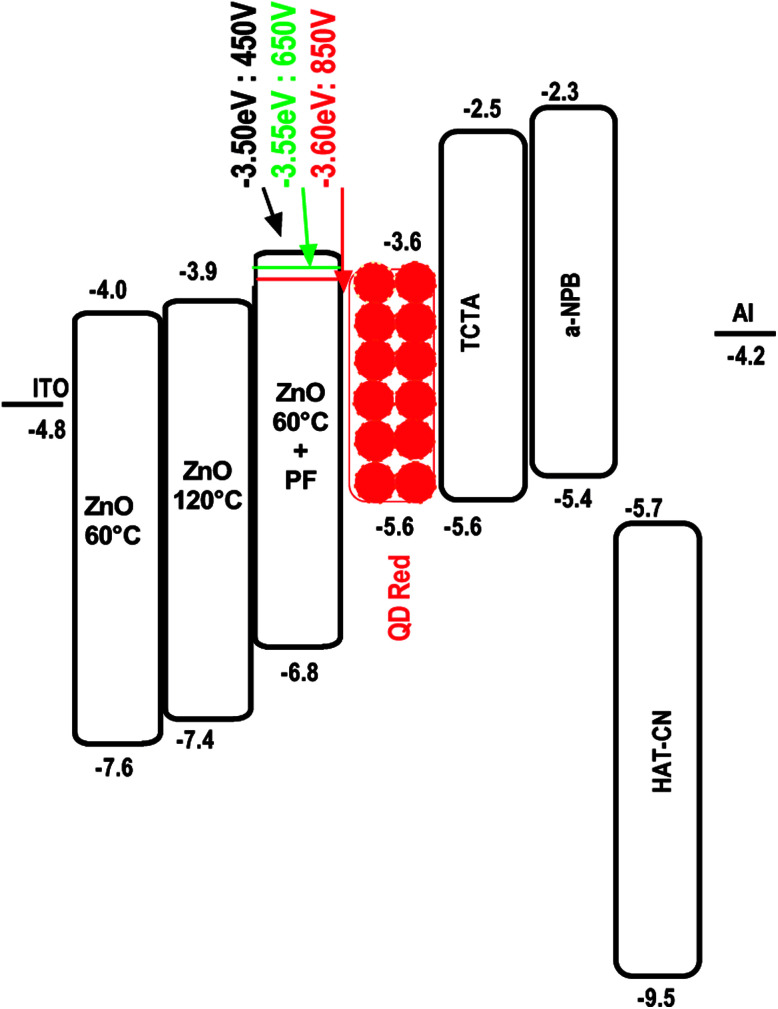
Energy level diagram depicting the HOMO–LUMO levels in relation to the CdSe/ZnS QD. TCTA acts as an electron blocker, α-NPB (hole transporter) and HAT-CN (hole injector).

The energy level diagrams for the devices Type, A, B and C are shown in Fig. 3.

## Results and discussion

### 1. Transmittance spectroscopy

**Fig. 4:**
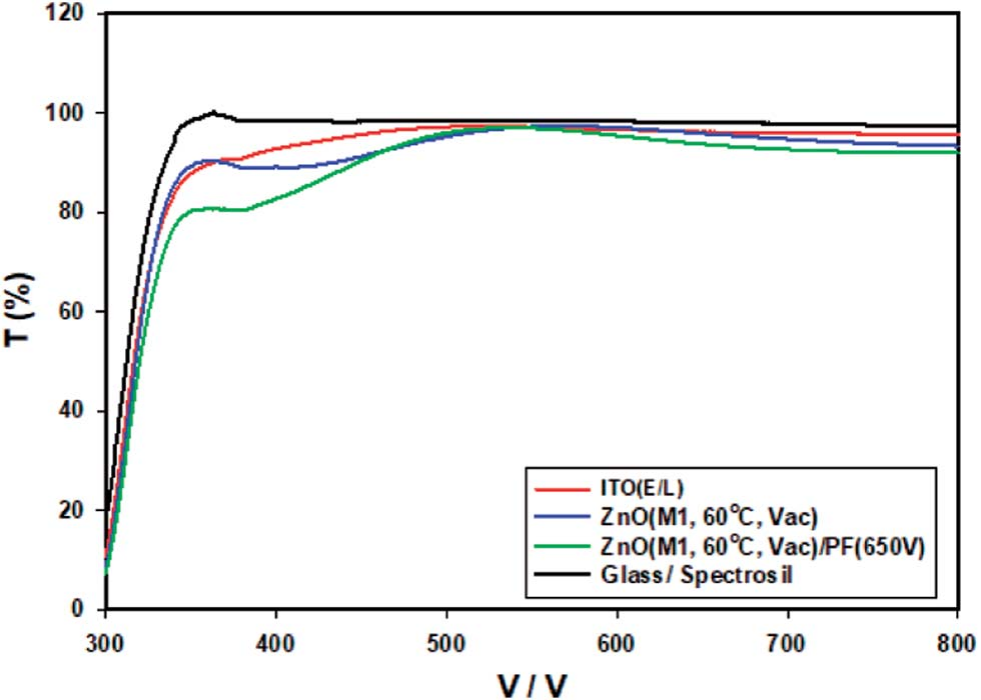
The transmittance spectra of glass, ITO/glass and ZnO-60 °C (vacuum) are compared with ZnO-60 °C + IPL (PF at 650 V).

Absorption and transmission spectroscopy of the thin films of thermally annealed ZnO films and IPL irradiated ZnO films were measured from 300 nm to 800 nm. The transmittance spectra of thermally annealed ZnO with photonically annealed ZnO are compared in Fig. 4.

The transmittance of the ZnO produced by IPL (PF) is significantly lower than that of ZnO-60 °C particularly in the blue region. In the 600–650 nm, the transmittance of ITO/Glass, ZnO-60 °C/Glass and ZnO-60 °C + PF (650 V) are 95%, 94% and 92% respectively. The band gaps were measured from the absorption edge to be 3.59 eV for ZnO-60 °C and 3.54 eV for ZnO-60 °C + PF. The band gaps for the ZnO films produced from sol–gel at 120 °C (vacuum) and 200 °C are 3.50 eV and 3.3 eV respectively.

### 2. X-ray diffraction

**Fig. 5:**
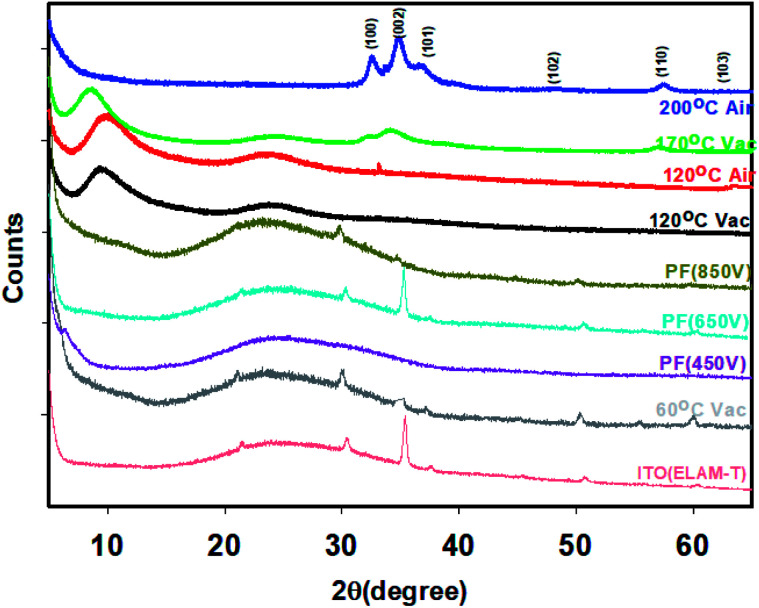
XRD of thin films of ZnO annealed under thermal and photonic annealing conditions. All the XRD on thermally annealed samples were on spectrosil (quartz) substrate except for the 60 °C (vacuum) which was on ITO/Glass substrates. All the IPL-PF samples are on ITO/Glass substrates.

The evolution of the XRD is shown in Fig. 5. The crystallinity (determined from the software provided by the manufacturer, known as Diffrac Suite Version 4 (Diffrac.eva)) increase as the temperature increases consistent with previous reports by other workers^39,40^ and is dependent on whether the films were annealed in vacuum or air. Air annealed samples have higher crystallinity as shown in the Table 2.

**Table 2:** Crystallinity of the ZnO thin films produced at different temperatures and environment

Temperature and condition of annealing	Crystallinity (%)
200 °C (air)	90.2
170 °C (vacuum)	63.0
120 °C (air)	88.6
120 °C (vacuum)	60.9
60 °C (vacuum)	15.7
350 V (Pulse Forge)	16.1
450 V (Pulse Forge)	17.2
650 V (Pulse Forge)	23.9
850 V (Pulse Forge)	24.0

XRD of the ZnO films annealed at 120 °C (vacuum) shows a reasonably large amount of crystallinity (60.9%). The films made at 60 °C (vacuum) has a very poor crystallinity of 15.7%. The crystallinity of the films annealed at 350 V, 450 V, 650 V and 850 V are 16.1, 17.2, 23.9 29.3 and 24.0 respectively. It is interesting to note that crystallinity of the film exposed to IPL at 350 V (air) itself exceeds that of 60 °C (vacuum). Further, the results show that higher the pulse voltage, pulse width and pulse time (energy input), higher is the crystallinity of the films which in turn increases the mobility.

### 3. Atomic force microscopy

**Fig. 6:**
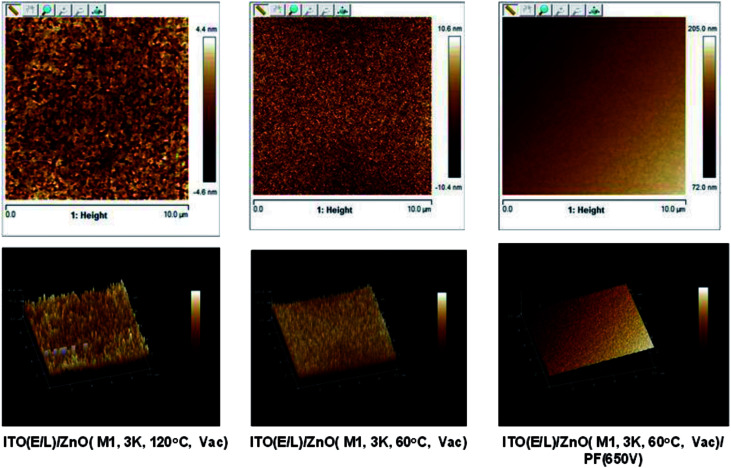
AFM images of ZnO-60 °C, ZnO-120 °C and ZnO-60 °C + IPL (Pulse Forge, 650 V).

AFM microscopy of ZnO films annealed at 120 °C, 60 °C and 60 °C + PF (650 V) is shown in Fig. 6. The topography of the films annealed at 120 °C (vac) has large features of irregularly shaped nodules (100–300 nm) whereas the films produced at 60 °C has much smaller features (10–50 nm) and that produced at 60 °C + PF (650 V, energy density 37.8 J cm^−2^) shows even smaller features (5–10 nm) and the surface is very smooth. The roughness data are presented in Table 3. The *R*_a_ values of the films grown at 120 °C (vacuum), 60 °C (vaccum) and 60 °C + PF (650 V) are 4.60, 3.4 and 1.19 nm respectively. The high smoothness of the film enables the quantum dots to produce pinhole free films.

**Table 3:** Roughness data over 10 μm × 10 μm area of ZnO-60 °C, ZnO-120 °C and ZnO-60 °C + IPL (Pulse Forge, 650 V) films

Parameter	ZnO (60 °C, vacuum)	ZnO (120 °C, vacuum)	ZnO (60 °C, vacuum) + IPL (PF, 650 V)
*R*_a_ (nm)	3.40	4.60	1.19
*R*_q_ (nm)	2.00	5.80	1.47
*R*_max_ (nm)	31.20	45.40	17.10

### 4. Contact angle measurements

**Fig. 7:**
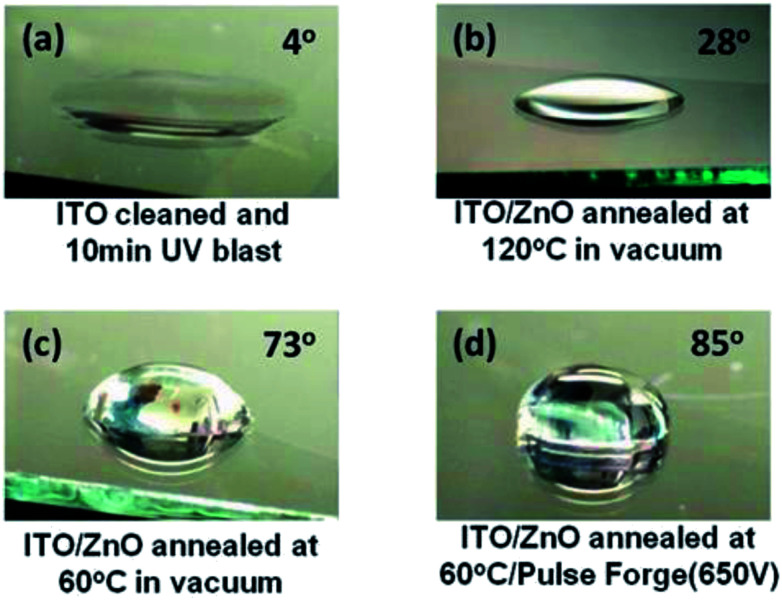
Water droplet on (a) ITO/glass (Ozone/UV cleaned), (b) ZnO (120 °C)/ITO/Glass, (c) ZnO (60 °C)/ITO/Glass and (d) ZnO (60 °C) + PF (650 V)/ITO/Glass.

Contact angle measurements were carried out with water droplets on ITO (cleaned and Ozone/UV treated) and the ZnO films annealed at 120 °C, 60 °C and 60 °C + IPL (PF) (Fig. 7) and the contact angles were found to be 4°, 28°, 73° and 85° indicating that the surfaces are becoming more and more hydrophobic. The increase in hydrophobicity is primarily due the surface topography and morphology (presence of different degrees of organics on the surface),^45–47^ crystal orientation^43^ oxygen vacancy and the surface roughness.^44^ We believe that the hydrophobic films are essential for the adhesion of the quantum dots onto the ZnO surface.

### 5. Electron only devices

**Fig. 8:**
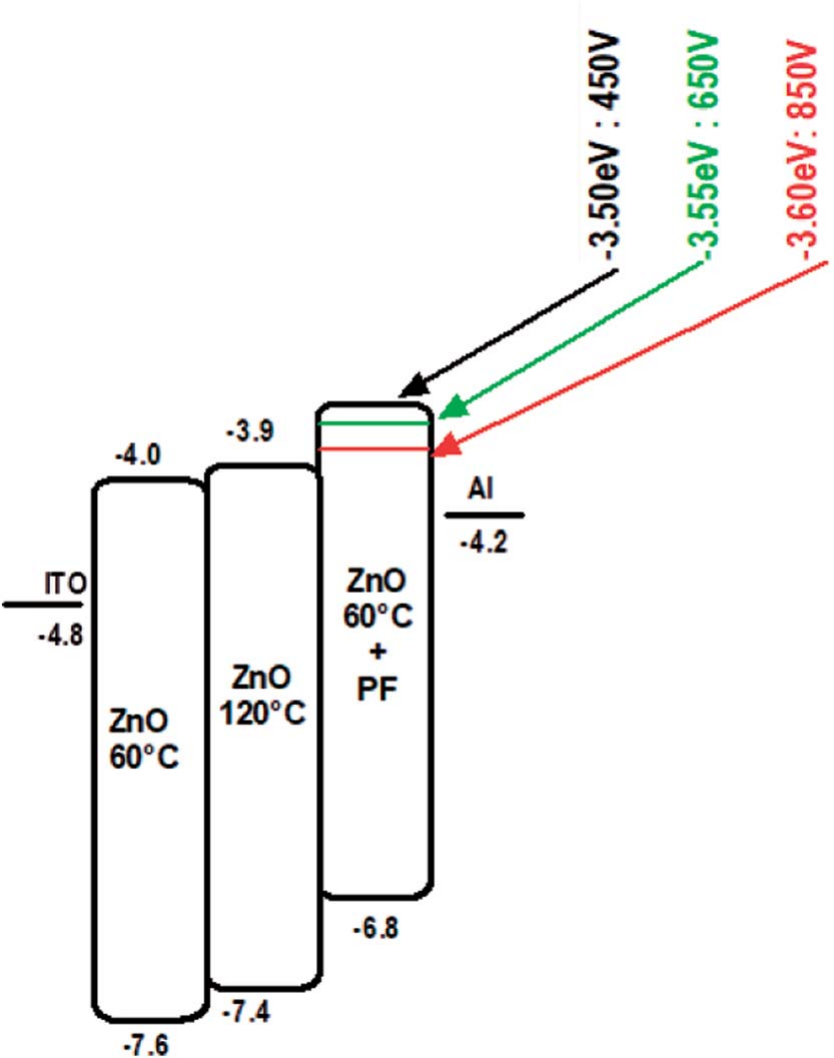
Energy level diagram for ZnO-60 °C, ZnO-120 °C and ZnO-60 °C + IPL (Pulse Forge, 650 V).

The electron only devices (ITO/Al/ZnO/Al) were made for ZnO-120 °C and ZnO-60 °C and ZnO-60 °C + IPL (PF-650 V) and the energy level diagram is shown in Fig. 8.

**Fig. 9:**
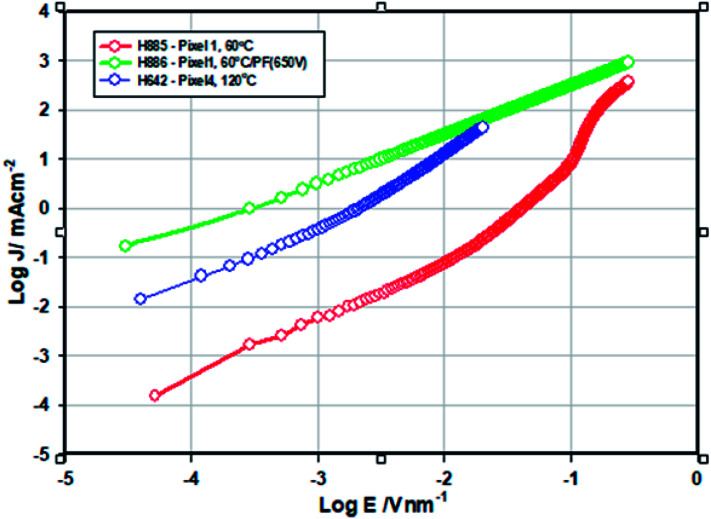
Log(current density (*J*/mA cm^−2^)) *vs.* log field (*E*/V nm^−1^) for ZnO-120 °C and ZnO-60 °C except for 60 °C + IPL (PF) based single layer devices (ITO/Al/ZnO/Al).

The current density *vs.* voltage (Fig. 9) of the electron only devices of the ZnO films −120 °C and ZnO-60 °C showed space charge limited current behaviour while ZnO-60 °C + IPL (PF) which showed ohmic behaviour under our experimental condition. As can be seen, the current density for any given field is highest for ZnO-60 °C + PF 650 V and the least for 60 °C (vacuum).

The mobility was calculated from the space charge region (*J vs. V*^2^) (See ref. 28 and 29). The conductivity of ZnO-60 °C + PF (650 V) is 625 fold higher than that of ZnO-60 °C (5.6 × 10^−10^ S cm^−1^) (Table 4). We attribute this increase in conductivity to the effective removal of the organics and the ligands and photonic doping.^10,25^ Similarly, the conductivity ZnO-120 °C (vac) is 53.6 times higher than that of ZnO-60 °C and this is attributed to the increased crystallinity (23.9% for ZnO-IPL *vs.* 15.7% for ZnO-60 °C), increased oxygen vacancy and the reduction in strain (increase in grain size).^42^

**Table 4:** Conductivity and mobility data of the ZnO films

Sample	Conductivity/S cm^−1^	Mobility/cm^2^ V^−1^ s^−1^
ZnO-120 °C	3.0 × 10^−8^	3.0 × 10^−5^
ZnO-60 °C	5.6 × 10^−10^	4.3 × 10^−8^
ZnO-60 °C + PF (650 V)	3.5 × 10^−7^	a

a No SCLC region and therefore mobility cannot be determined as the conduction was found to ohmic.

The mobility of ZnO-120 °C (3 × 10^−5^ cm^2^ V^−1^ s^−1^) is 7 times higher than that of the ZnO-60 °C. This is attributed to do a significant amount residual organic ligands (incomplete removal) left behind in the film. We speculate that the mobility of ZnO-60 °C + PF-650 V would be higher than the ZnO-60 °C on the basis of the turn on voltage of the QLED devices made from this (See later).

**Fig. 10:**
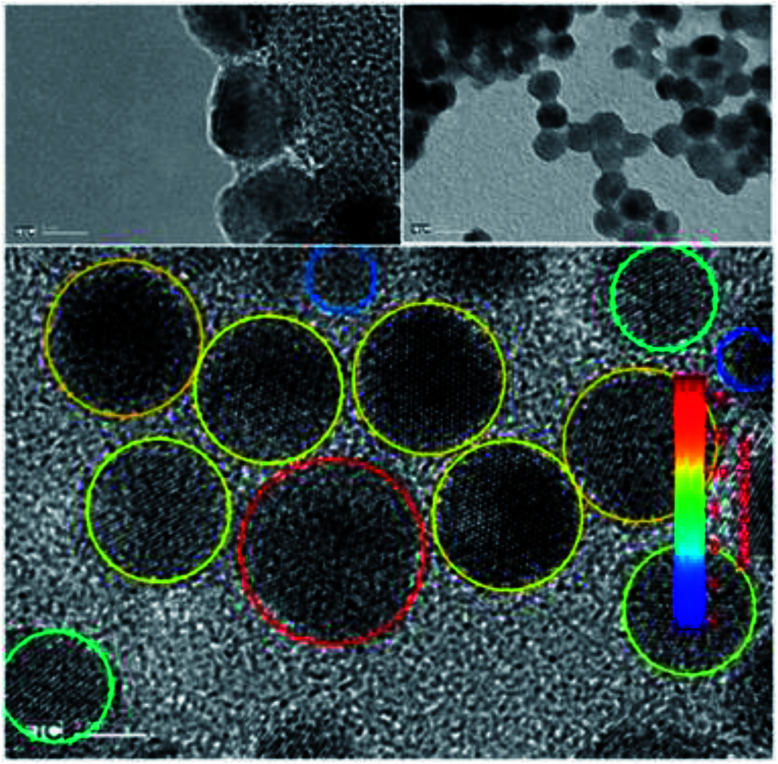
TEM image of the red quantum dots employed in this study.

Transmission electron microscopy of the quantum dots reveals (Fig. 10) shows an average particle of size of 8.9 ± 2.6 nm, but the particle ranging from 4 nm to 13 nm have been found.

### 6. Electroluminescent devices

**Fig. 11:**
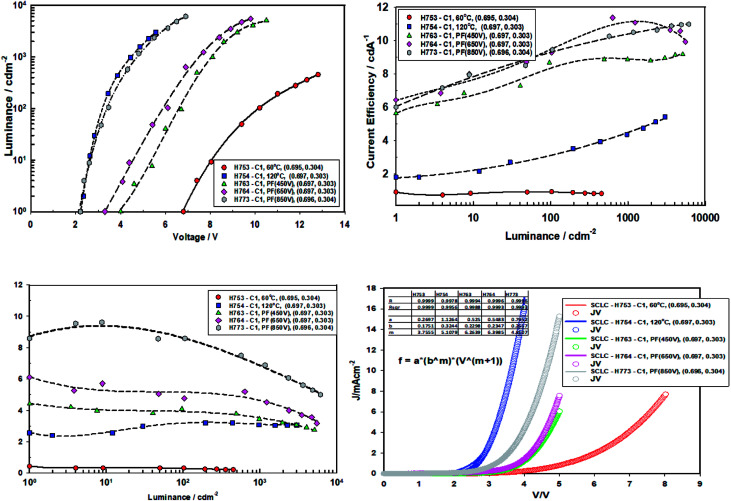
The electroluminescent spectra of devices Type A, Type B and Type C (ITO(E/L) ZnO (M1, 3 K, *X* °C, 30 min, vac)/IPL-PF(Y)/QD-R(BT, 3 K, 80 °C, 20 min, vac)/TCTA(10)/a-NPB(10)/HAT-CN(10)/Al) comprising ZnO (120 °C), ZnO (60 °C), ZnO (60 °C) + PF (450 V), ZnO (60 °C) + PF (650 V) and ZnO (60 °C) + PF (850 V).

**Fig. 12:**
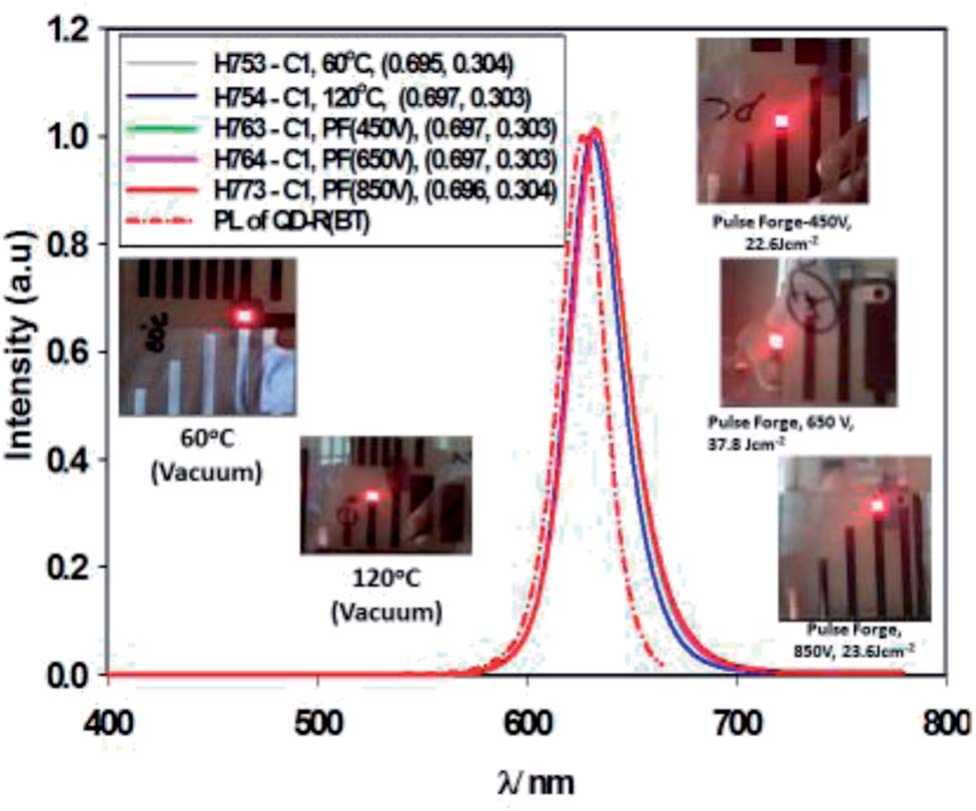
Photoluminescent spectra of the QD compared with the electroluminescent spectra of devices comprising ZnO (60 °C), ZnO (60 °C) + PF (450 V), ZnO (60 °C) + PF (650 V) and ZnO (60 °C) + PF (850 V). Inset, Electroluminescent devices.

Fig. 11 gives the performance plots for Set A (ITO/ZnO (NP) (40 nm, Brunel-M1, 60 °C)/Red-QD-X (25 nm)/TCTA (10 nm)/HTL (α-NPB (20 nm)/HATCN (10 nm)/Al)), Set B (ITO/ZnO (NP) (40 nm, Brunel-M1, 120 °C)/Red-QD-X (25 nm)/TCTA (10 nm)/HTL (α-NPB (20 nm)/HATCN (10 nm)/Al)) and Set C (ITO/ZnO (NP) (40 nm, Brunel-M1, 60 °C) and Intense Light Pulse (ILP-PF)/Red-QD-X (25 nm)/TCTA (10 nm)/HTL (α-NPB (20 nm)/HATCN (10 nm)/Al)) immediately after fabrication. Table 5 gives a summary of the device performance. The PL and the EL spectra of the devices are shown in Fig. 12. There is a small bathochromic shift for the EL of approximately 3–5 nm as has been observed with most QLEDs.^26^

**Table 5:** Device performance immediately after fabrication (*t* = 0)^a^

ID	Condition	*V*_T_ (V)	@1000 cd m^−2^				Maximum		*m*
			*V*_D_ (V)	C/E (cd A^−1^)	P/E (lm W^−1^)	CIE (*x*, *y*)	C/E (cd A^−1^)	P/E (lm W^−1^)	
H753	60 °C	6.8 ± 0.1	12.8 ± 0.1	0.8 ± 0.1	0.20 ± 0.1	(0.695, 0.304)	0.9	0.4	3.76
H754	120 °C	2.2 ± 0.1	4.4 ± 0.1	4.4 ± 0.1	3.1 ± 0.1	(0.696, 0.303)	5.4	3.2	5.11
H763	PF (450 V)	4.0 ± 0.1	8.1 ± 0.1	9.0 ± 0.1	3.5 ± 0.1	(0.697, 0.303)	9	4.5	6.26
H764	PF (650 V)	3.3 ± 0.1	7.4 ± 0.1	11.0 ± 0.1	4.7 ± 0.1	(0.697, 0.303)	11	6	6.40
H773	PF (850 V)	2.2 ± 0.1	4.6 ± 0.1	11.0 ± 0.1	7.5 ± 0.1	(0.696, 0.304)	11	10	4.81

a *V*_t_: turn-on voltage (1 cd m^−2^), *V*_d_: drive voltage to give 1000 cd m^−2^, C/E: current efficiency, P/E: power efficiency. Value *m* is *J*_d_ = *ab*^*m*^*V*^*m*+1^ from the current density *vs.* voltage plots.

It is clear from the Fig. 11 that the devices fabricated with ZnO-60 °C (vacuum) have poorest efficiency, followed by ZnO-120 °C. What is surprising is that all the devices fabricated with ZnO irradiated with intense pulse light (PF–PF) show significantly improved performance. The turn-on voltage of the Set A (ZnO-60 °C) devices is 6.8 V and that of the Set B (ZnO-120 °C) is 2.2 V. This can be explained on the basis of the poor mobility (698 fold lower mobility) and conductivity (nearly 53.4 fold lower) of the ZnO-60 °C compared to ZnO-120 °C. However, devices made with ZnO-IPL (PF) (devices C) yield turn on voltages of 4.0, 3.3 and 2.2 V for the intense light pulses at 450 V, 650 V and 850 V respectively. Indeed, the devices made at IPL (PF) of 850 V shows identical turn-on voltage to the device Type B (ZnO-120 °C). It is important to note that the IPL (Pulse Forge) was carried out at the room temperature in the ambient atmosphere.

Both the current efficiency and power efficiency increases both with the annealing temperature (Type B *vs.* Type A) and the pulse voltage and the energy density of the IPL (PF). There is a dramatic increase in efficiency when Type A (60 °C, vacuum) devices are compared with Type C (ZnO-60 °C + PF). For example, the current efficiency at ZnO-60 °C-850 V pulse is 11 cd A^−1^ compared to un-irradiated case, 0.8 cd A^−1^. Similarly, the power efficiency is 37.5 fold higher (7.5 lm W^−1^ for ZnO-60 °C-850 V *vs.* 0.2 lm W^−1^ for ZnO-60 °C).

The current injection is highest for the for the ZnO-120 °C owing to its high mobility (3 × 10^−5^ cm^2^ V^−1^ s^−1^) and the poor current injection from ZnO-60 °C is due to the poor mobility of 4.3 × 10^−8^ cm^2^ V^−1^ s^−1^. The current injection for the ZnO-pulse forged film follows the order ZnO-450 V < ZnO-650 V < ZnO-850 V as the conductivity increases with voltage/pulse width and energy input.

The current density *vs.* voltage were fitted to the space charge equation^27–29^*J*_d_ (current density) = *ab*^*m*^*V*^*m*+1^

The *m* values are shown in Table 5. The *m* values follow the order ZnO-60 °C (*m* = 3.76) < ZnO-PF-850 V (*m* = 4.81) < ZnO-120 °C (*m* = 5.11) < 450 V (*m* = 6.26) < 650 V (6.40). Higher the *m* values greater is the dual carrier injection which leads to higher efficiency.^28,29,41^ It is worthwhile to note that the devices with ZnO-650 V received highest energy (energy density, 37.8 J cm^−2^), followed by ZnO-850 V (23.6 J cm^−2^) and ZnO-450 V (22.6 J cm^−2^).

### 7. Thermal aging (baking of the devices)

It has been demonstrated by Archarya^30^ as well as Kathirgamanathan *et al.*^26^ that the performance of the QLEDS can be improved by thermal annealing (aging) of the finished devices. We wanted to explore if this is the case for IPL (pulse forged) annealed ZnO based devices (Type C).

**Fig. 13:**
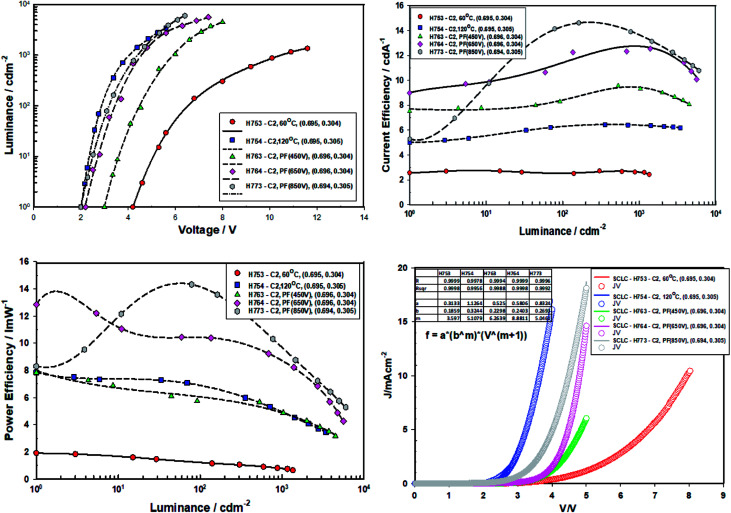
Performance data of devices comprising ZnO (120 °C), ZnO (60 °C), ZnO (60 °C) + PF (450 V), ZnO (60 °C) + PF (650 V) and ZnO (60 °C) + PF (850 V) after 10 minutes of aging at 40 °C.

**Fig. 14:**
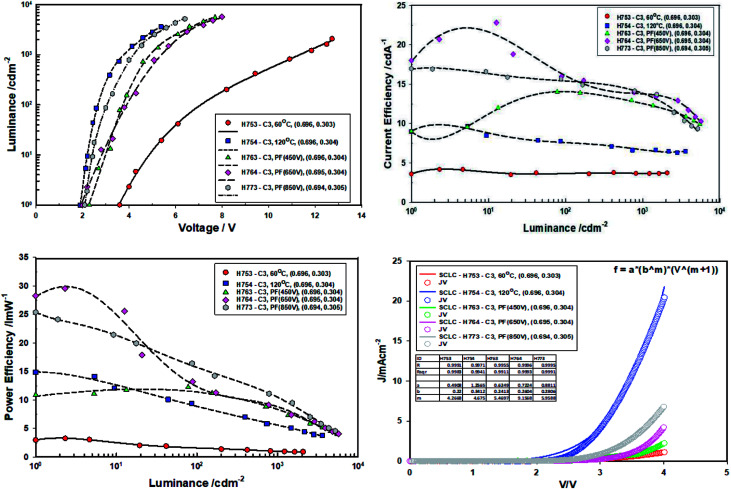
Performance data of devices comprising ZnO (120 °C), ZnO (60 °C), ZnO (60 °C) + PF (450 V), ZnO (60 °C) + PF (650 V) and ZnO (60 °C) + PF (850 V) after aging the devices at 40 °C for 24 hours.

The devices (Type A, B and C) were then heated and maintained at 40 °C under nitrogen atmosphere for 10 minutes and the device performance measured (Fig. 13 and Table 6), then for 24 hours (Fig. 14 and Table 7) under nitrogen at 40 °C and performance measured.

**Table 6:** Performance of device Types A, B and C after heating at 40 °C for 10 minutes^a^

ID	Condition	*V*_T_ (V)	@1000 cd m^−2^				Maximum		*m*
			*V*_D_ (V)	C/E (cd A^−1^)	P/E (lm W^−1^)	CIE (*x*, *y*)	C/E (cd A^−1^)	P/E (lm W^−1^)	
H753	60 °C	4.2 ± 0.1	10.2 ± 0.1	2.6 ± 0.1	0.8 ± 0.1	(0.695, 0.304)	2.7	1.9	3.60
H754	120 °C	2.0 ± 0.1	4.2 ± 0.1	6.4 ± 0.1	4.8 ± 0.1	(0.696, 0.304)	6.4	7.5	5.11
H763	PF (450 V)	3.0 ± 0.1	6.1 ± 0.1	9.0 ± 0.1	4.6 ± 0.1	(0.696, 0.304)	10	8	6.26
H764	PF (650 V)	2.2 ± 0.1	4.4 ± 0.1	11.0 ± 0.1	7.9 ± 0.1	(0.696, 0.304)	12	9	8.88
H773	PF (850 V)	2.0 ± 0.1	4.2 ± 0.1	14.0 ± 0.1	10.5 ± 0.1	(0.694, 0.305)	15	14	5.05

a *V*_t_: turn-on voltage (1 cd m^−2^), *V*_d_: drive voltage to give 1000 cd m^−2^, C/E: current efficiency, P/E: power efficiency. Value *m* is *J*_d_ = *ab*^*m*^*V*^*m*+1^ from the current density *vs.* voltage plots.

**Table 7:** Performance of device Types A, B and C after heating at 40 °C for 24 hours^a^

ID	Condition	*V*_T_ (V)	@1000 cd m^−2^				Maximum		*m*
			*V*_D_ (V)	C/E (cd A^−1^)	P/E (lm W^−1^)	CIE (*x*, *y*)	C/E (cd A^−1^)	P/E (lm W^−1^)	
H753	60 °C	3.8 ± 0.1	10.9 ± 0.1	3.7 ± 0.1	1.1 ± 0.1	(0.696, 0.303)	4.2	3.3	4.27
H754	120 °C	1.9 ± 0.1	3.8 ± 0.1	6.6 ± 0.1	5.5 ± 0.1	(0.696, 0.304)	10	15	4.68
H763	PF (450 V)	2.6 ± 0.1	4.6 ± 0.1	13 ± 0.1	8.9 ± 0.1	(0.696, 0.304)	14	12	5.47
H764	PF (650 V)	2.0 ± 0.1	4.8 ± 0.1	14 ± 0.1	9.2 ± 0.1	(0.696, 0.304)	23	30	9.15
H773	PF (850 V)	2.1 ± 0.1	4.0 ± 0.1	14 ± 0.1	11.0 ± 0.1	(0.694, 0.305)	18	26	5.97

a *V*_t_: turn-on voltage (1 cd m^−2^), *V*_d_: drive voltage to give 1000 cd m^−2^, C/E: current efficiency, P/E: power efficiency. Value *m* is *J*_d_ = *ab*^*m*^*V*^*m*+1^ from the current density *vs.* voltage plots.

When the devices were thermally aged (baked), the efficiency of all the devices increased significantly and turn-on voltage and the drive voltage reduced. The turn on-voltage and the drive voltage of pulse forged (ILP) devices approached that of the devices made from ZnO-120 °C. It is exciting to note that devices made from ZnO-60 °C-PF (room temperature) have the same turn-on voltage and the drive voltage as those devices made with ZnO-120 °C. However, the current efficiency ZnO-60 °C-PF -850 V (RT) (14 cd A^−1^) and is nearly double that of ZnO-120 °C (6.6 cd A^−1^) and the power efficiency is actually doubled (from 5.5 lm W^−1^ to 11 lm W^−1^) if one considers the devices aged for 24 hours.

What is most remarkable is that the enhancement of efficiencies of devices Type C when compared to devices Type A. The current efficiency increases by 3.78 fold and the power efficiency by 10 fold for the case of ZnO-60 °C + PF850 V as far as the devices baked for 24 hours are concerned. Furthermore, the devices where the ZnO film was pulsed at 650 V yielded a maximum current efficiency of 23 cd A^−1^ and a maximum power efficiency of 30 lm W^−1^. The results are summarised in Tables 6 and 7.

The *m* value for the devices aged for 10 minutes at 40 °C follow the same order as the virgin devices, namely ZnO-60 °C (*m* = 3.60) < ZnO-PF-850 V (*m* = 5.05) < ZnO-120 °C (*m* = 5.11) < 450 V (*m* = 6.26) < 650 V (*m* = 8.88). There is steep increase in the *m* value for ZnO-60 °C + PF 650 V (*m* = 8.88) on going for virgin devices to the devices (*m* = 6.40) annealed for 10 minutes. Such a high value of *m* reflects more efficient dual carrier injection and is reflected in the significant increase (68%) in the power efficiency of the ZnO-60 °C + PF 650 V devices.

**Fig. 15:**
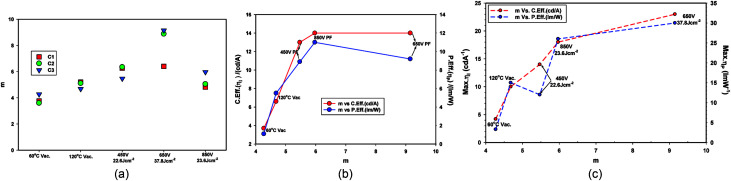
(a) *m* value *vs.* thermal or IPL treatment condition for devices after 24 hour baking at 40 °C (left). (b) Current efficiency (cd A^−1^) and power efficiency (lm W^−1^) *vs. m* at 1000 cd m^−2^ (middle). (c) Maximum current efficiency and maximum power efficiency *vs. m* (right).

Table 8 compares the *m* values for the devices Set A (ZnO-60 °C (vacuum)), Set B (ZnO-120 °C (vacuum)) and Set C (ZnO-60 °C (vacuum) + PF). As explained earlier, higher the *m* values, more dual carrier injection it is. Dual carrier injection is a prerequisite for light emission. Fig. 15 illustrates the changes on *m* values under different conditions. The *m* value of ZnO-60 C + PF 650 V (37.8 J cm^−2^) devices yield the highest *m* value and the second highest is for ZnO-60 C + PF 850 V (23.6 J cm^−2^) devices. It appears that once certain level intensity of light is reached, then the determining factor is the total energy input as far as the maximum (peak) efficiencies are concerned.

**Table 8:** *m* values from the SCLC (*J*_d_ = *ab*^*m*^*V*^*m*+1^ from the current density *vs.* voltage plots)

ID	Condition	*m* value (time = 0)	*m* value (aging time 10 minutes)	*m* value (aging time 24 hours)	Comments
H753	60 °C	3.76 ± 0.20	3.60 ± 0.20	4.27 ± 0.20	No significant change upto 10 minute baking. Slight increase with baking
H754	120 °C	5.10 ± 0.20	5.11 ± 0.20	4.68 ± 0.20	No change upto 10 minute baking. No slight decrease with baking for 24 hours
H763	PF (450 V) (energy density, 22.6 J cm^−2^)	6.26 ± 0.20	6.26 ± 0.20	5.47 ± 0.20	No change upto 10 minute baking. Slight decrease with baking
H764	PF (650 V) (energy density, 37.8 J cm^−2^)	6.40 ± 0.20	8.88 ± 0.20	9.15 ± 0.20	Highest *m* value, significant increase on baking
H773	PF (850 V) (energy density, 23.6 J cm^−2^)	4.81 ± 0.20	5.05 ± 0.20	5.97 ± 0.20	No significant change upto 10 minute baking. Increase in *m* value on baking

### 8. Life time measurements

**Fig. 16:**
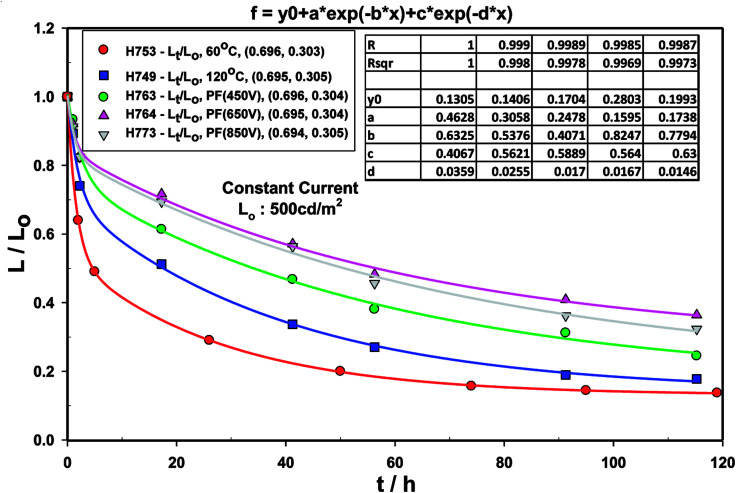
Luminance with time decay curve for devices comprising ZnO (120 °C), ZnO (60 °C), ZnO (60 °C) + PF (450 V), ZnO (60 °C) + PF (650 V) and ZnO (60 °C) + PF (850 V).

The life-time of the Type A, B and C devices were measured at an initial luminance of 500 cd m^−2^ under constant current (Fig. 16) and the half-life is summarised in Table 9.

**Table 9:** Half-life of devices comprising ZnO (120 °C), ZnO (60 °C), ZnO (60 °C) + PF (450 V), ZnO (60 °C) + PF (650 V) and ZnO (60 °C) + PF (850 V) measured at an initial luminance of 500 cd m^−2^

Device ID	Device structure	Life time (*t*_1/2_)/hours
H753	ZnO-60 °C (Type A)	10 ± 2
H754	ZnO-120 °C (Type B)	18 ± 2
H763	ZnO-450 V (PF)-Type C	35 ± 2
H764	ZnO-650 V (PF)-Type C	57 ± 2
H773	ZnO-850 V (PF)-Type C	55 ± 2

The normalised luminance (*L*/*L*_o_) was fitted to the *L*/*L*_o_ = *α* + *a* exp(−*k*_1_*t*) + *c* exp(−*k*_2_*t*) where *L* is the luminance at any time *t*, *L*_o_ is initial luminance, *α*, *a*, *c*, *k*_1_ and *k*_2_ are constants. Good fit was obtained for all the devices as has been seen for most OLED devices. This indicates that the decay in luminance is biphasic.

It is clear that the devices made with ZnO-PF as the etl/eil have nearly 5.7 longer life time than the control (*i.e.* No IPL (PF)). We believe that intense pulsed ZnO has considerably higher stability than the ZnO produced by simple thermal annealing, thus has much higher electrical and electrochemical stability and may be of general applicability. We also believe that IPL (Pulse Forge) treated ZnO leads to much more balanced carrier injection leading to longer life time.

**Fig. 17:**
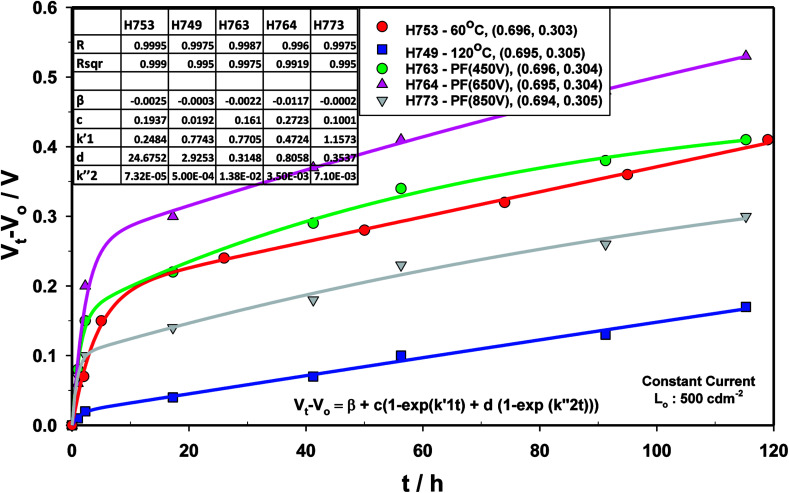
Drift voltage for devices comprising ZnO (120 °C), ZnO (60 °C), ZnO (60 °C) + PF (450 V), ZnO (60 °C) + PF (650 V) and ZnO (60 °C) + PF (850 V).

The voltage drift (Fig. 17) was fitted to the equation 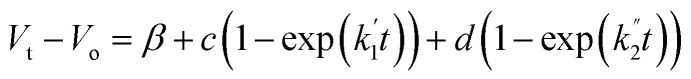 where *V*_t_ is the voltage at any time *t*, *V*_o_ is the initial voltage, *β*, *c*, *d*, 
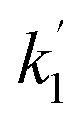
 and 
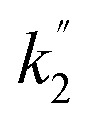
 are constants. Again, good fit was obtained for all the devices. The voltage drift therefore appears to be biphasic, similar to luminance decay of OLEDs.

## Conclusion

**Fig. 18:**
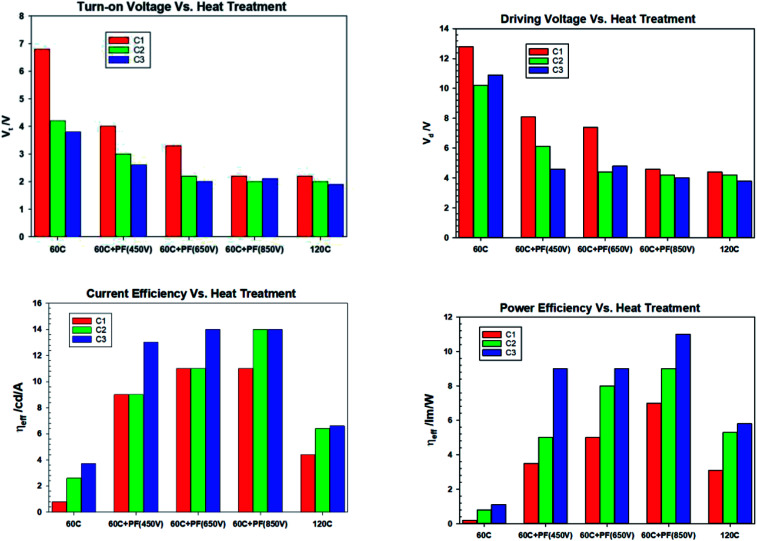
Turn-on voltage, drive voltage at 1000 cd m^−2^, current efficiency at 1000 cd m^−2^ and power efficiency at 1000 cd m^−2^*vs.* baking time for devices comprising ZnO (120 °C), ZnO (60 °C), ZnO (60 °C) + PF (450 V), ZnO (60 °C) + PF (650 V) and ZnO (60 °C) + PF (850 V). C1: *t* = 0, C2 = 10 minutes, C3: 24 hours.

We have demonstrated that the intense pulse light (IPL/Pulse Forge) can produce novel surface characteristics, surface modification (wetting characteristics) and potentially bulk modification (*e.g.* electrical conductivity). ZnO films subjected IPL becomes more conductive and hydrophobic thus enabling enhanced electron injection and improved wetting by non-aqueous solution of quantum dots, namely toluene in this investigation. We believe that the intense pulse light irradiation of metal oxides to modify the surface characteristics of general applicability and thermal aging of the devices dramatically increase the efficiency of the QD electroluminescent devices. The reduction in turn-on and drive voltages, increase in efficiency with pulse voltage/light energy input as well as thermal aging (baking) of the devices are shown in the Fig. 18 below:

Fig. 18 illustrates that, in general, the IPL (PF) annealed ZnO devices show significantly high efficiency and longer life time than the identical devices fabricated with thermally annealed ZnO.

**Fig. 19:**
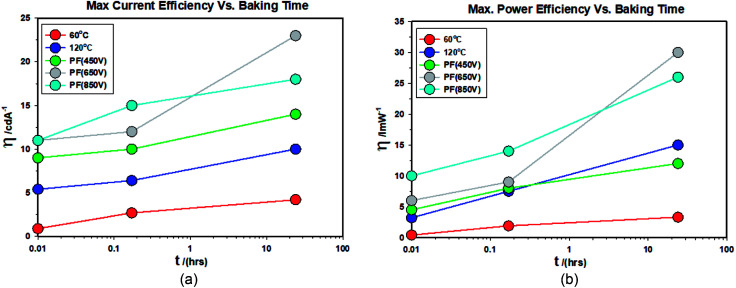
(a) Maximum current efficiency (left) and (b) maximum power efficiency (right) at 1000 cd m^−2^ for devices comprising ZnO (120 °C), ZnO (60 °C), ZnO (60 °C) + PF (450 V), ZnO (60 °C) + PF (650 V) and ZnO (60 °C) + PF (850 V). C1: *t* = 0, C2 = 10 minutes, C3: 24 hours.

In summary,

(I) The turn-on voltage of IPL-PF-650 V and 850 V devices is identical to ZnO-120 °C considering that the ZnO films were dried at 60 °C and pulse forged in air at room temperature.

(II) The drive voltage at 1000 cd m^−2^ also decreases with PF voltage. The drive voltage for PF-850 V is as same as ZnO-120 °C.

(III) The current efficiency increases with PF voltage (and light energy density) and is significantly higher than ZnO-120 °C at all luminance levels tested.

(IV) The power efficiency increases with PF voltage (and light energy density) and is significantly higher than ZnO-120 °C at all luminance levels tested.

(V) Fig. 19(a) shows the changes in the maximum current efficiency *vs.* baking time of the finished devices. They all increase with the baking time and the ZnO-PF-650 V devices show much higher enhancement than ZnO-PF-850 V for 24 hour baking at 40 °C. This is presumably due to the higher energy used for the 650 V (37.8 J cm^−2^) as opposed to 23.6 J cm^−2^ employed for the ZnO-PF-850 V.

(VI) Fig. 19(b) shows the changes in the maximum power efficiency *vs.* baking time of the finished devices. They all increase with the baking time and the ZnO-PF-650 V devices show much higher enhancement than ZnO-PF-850 V for 24 hour baking at 40 °C. This is presumably due to the higher energy used for the 650 V (37.8 J cm^−2^) as opposed to 23.6 J cm^−2^ employed for the ZnO-PF-850 V.

It is interesting to note that baking of the IPL-PF devices further improves the efficiency further and offers a general method to increase the performance of QLEDs.

## Conflicts of interest

There are no conflicts.

## Supplementary Material
